# Deceased Donor Characteristics and Kidney Transplant Outcomes

**DOI:** 10.3389/ti.2022.10482

**Published:** 2022-08-25

**Authors:** Adnan Sharif

**Affiliations:** ^1^ Department of Nephrology and Transplantation, University Hospitals Birmingham, Queen Elizabeth Hospital, Birmingham, United Kingdom; ^2^ Institute of Immunology and Immunotherapy, University of Birmingham, Birmingham, United Kingdom

**Keywords:** mortality, kidney transplant, graft loss, deceased donor, discard, kidney failure

## Abstract

Kidney transplantation is the therapy of choice for people living with kidney failure who are suitable for surgery. However, the disparity between supply versus demand for organs means many either die or are removed from the waiting-list before receiving a kidney allograft. Reducing unnecessary discard of deceased donor kidneys is important to maximize utilization of a scarce and valuable resource but requires nuanced decision-making. Accepting kidneys from deceased donors with heterogenous characteristics for waitlisted kidney transplant candidates, often in the context of time-pressured decision-making, requires an understanding of the association between donor characteristics and kidney transplant outcomes. Deceased donor clinical factors can impact patient and/or kidney allograft survival but risk-versus-benefit deliberation must be balanced against the morbidity and mortality associated with remaining on the waiting-list. In this article, the association between deceased kidney donor characteristics and post kidney transplant outcomes for the recipient are reviewed. While translating this evidence to individual kidney transplant candidates is a challenge, emerging strategies to improve this process will be discussed. Fundamentally, tools and guidelines to inform decision-making when considering deceased donor kidney offers will be valuable to both professionals and patients.

## Introduction

**FIGURE 1:**
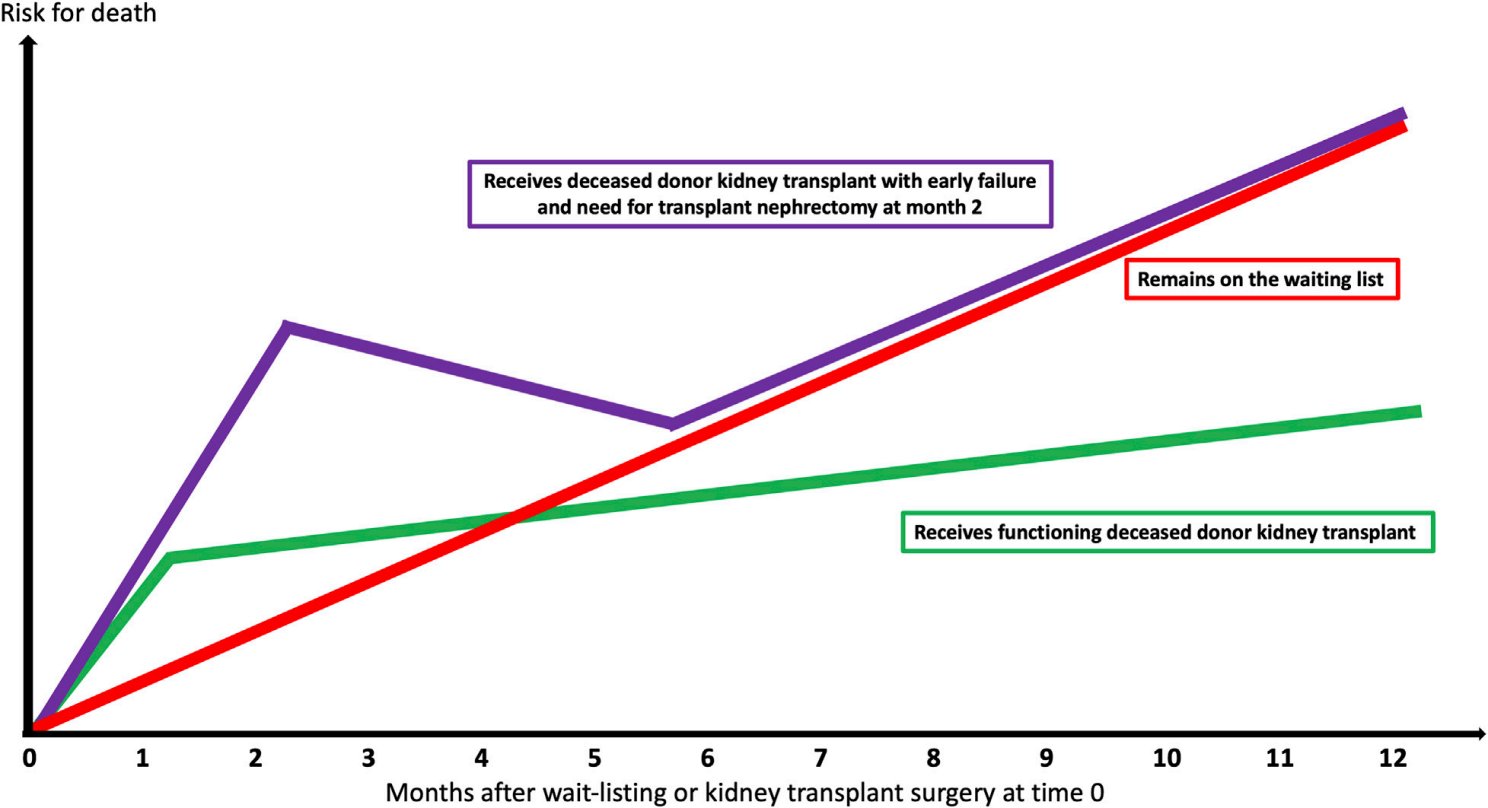
Survival probabilities based upon deceased donor kidney transplant success, failure, and remaining on the waiting-list.

**FIGURE 2:**
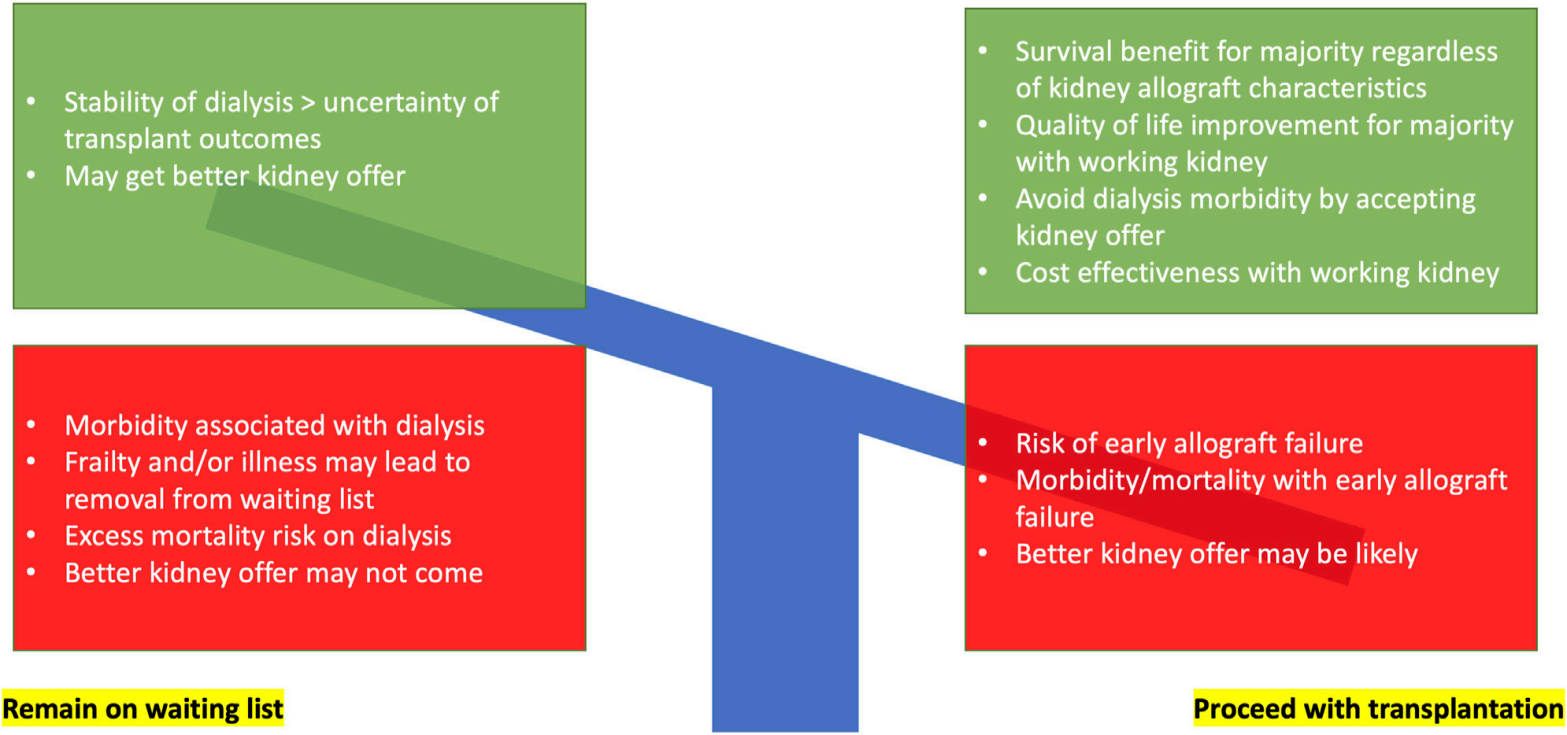
Decision-making for waitlisted kidney failure individuals with a deceased donor kidney offer balancing risk, benefit, and uncertainty.

Kidney transplantation is the treatment modality of choice for kidney failure patients deemed fit enough for surgery. While successful kidney transplantation lowers both cardiovascular (1) and all-cause mortality (2,3), and provides better quality of life and cost-effectiveness in most scenarios (4), kidney transplant failure and return to dialysis is associated with heightened risk for mortality over-and-above transplant-naïve waitlisted dialysis patients (see Figure 1) (5,6). Therefore, personalizing use of deceased donors for individual waitlisted kidney transplant candidates at the most appropriate time is challenging (see Figure 2) (7).

These factors partly explain unnecessary kidney discards. Mohan et al. observed 17.3% of procured kidneys in the United States between 2000–2015 were discarded, despite partner kidneys of unilaterally discarded kidneys experiencing 1-year death-censored graft survival rates >90% (8). Over 80% of kidney discard rates can be explained by the broadening donor pool and unexplained residual factors (9). Organ discard rates in European countries are lower than the United States (10), although donor characteristics differ (e.g., more opioid-related deaths in the United States) (11). If deceased donor kidney acceptance in the United States mirrored the French model (discard rate 17.9% versus 9.1% respectively, *p* < 0.001), then utilization of discarded kidneys (*n* = 17,435) could generate 132,445 allograft life-years (12). This is important as declined kidney offers are not benign events. Husain et al., in a cohort study analyzing 280,041 wait-listed kidney transplant candidates in the United States, observed approximately 30% of candidates receiving at least one deceased donor offer declined on their behalf eventually died or were removed from the waiting-list before receiving a kidney allograft (13). Apart from clinical benefits, transplantation using kidneys of any quality is cost-effective versus remaining on the waiting-list (14).

In view of increasing marginality of kidneys procured from deceased donors (15,16), which contributes to sub-optimal organ utilization, informed decision-making to accept kidney offers for individual kidney transplant candidates must be the objective. While organ allocation systems continue to evolve (17), which impact upon utilization, the aim of this review is to summarize published evidence regarding kidney transplant outcomes associated with deceased kidney donor characteristics. Translating such data into decision-making pathways is a clinical challenge and emerging ways to foster better organ utilization are discussed.

## Donor Clinical Factors

### Expanded Criteria Donor

An ECD is one who, at the time of death, is aged ≥60 years or aged 50–59 years with any two the following three criteria: 1) cause of death is cerebrovascular accident, 2) pre-existing history of systemic hypertension, and 3) terminal serum creatinine >1.5 mg/dl. The criteria for defining ECD was based on the presence of variables that historically increased the risk for graft failure by 70% compared with a standard criteria donor (SCD) kidney (18).

Previous systematic reviews suggested ECD kidneys should not be offered to younger (aged <40 years) kidney transplant candidates or those undergoing re-transplantation (19). ECD kidneys may be better prioritized for older recipients by ignoring immunology-based allocation. Using this strategy, the Eurotransplant Senior program have shown favorable 5-year outcomes using ECD kidneys in older recipients (20).

However, recent analyses support broadening access with careful risk stratification. Querard et al. conducted a systematic review and meta-analysis of 32 studies comparing survival outcomes between SCD and ECD kidneys (21). Pooled 5-year patient survival probabilities were 78.4% versus 86.4% in ECD versus SCD recipients respectively. A significant difference in mortality was observed comparing North American and European studies, with 5-year pooled patient survival closer in European studies (ECD versus SCD; 85.3% versus 90.3% respectively) than in North American studies (ECD versus SCD, 73.4% versus 83.6% respectively). The corresponding pooled RR was estimated at 1.50 (95% CI 0.50–3.43) for the European studies versus 1.62 (95% CI 1.18–2.22) for the North American studies. Similar effect sizes were seen with regards to death-censored graft survival.

ECD kidney allograft survival may be improved in the absence of circulating donor-specific antibody (*p* < 0.001) and CIT <12 h (*p* = 0.030) according to a French study (22). Optimal utilization of ECD kidneys may also be stratified by recipient age, with studies suggesting recipients aged ≥60 years (23) or ≥65 years (24) be prioritized. However, while a 1.75-fold (95% CI 1.53–2.00, *p* < 0.0001) increased risk for graft failure using ECD versus SCD kidneys was observed in one study, population-average effect using propensity scores with 10-year follow-up highlighted a minimal absolute effect of only 8 months (95% CI 2–14 months) quicker time to graft failure attributed to ECD kidneys (24). Therefore, the absolute risk difference between SCD and ECD kidneys in the long-term may be marginal when compared to remaining on the waiting-list.

### Donation After Cardiac Death

DCD refers to a donor who does not meet the criteria for donation after brain death (DBD) but in whom cardiac standstill or cessation of cardiac function occurred before organs were procured, with cessation of cardiac function initiated deliberately (controlled) or occurring spontaneously (uncontrolled) (18).

Data from the United Kingdom, examining outcomes in adult recipients receiving a deceased donor kidney transplant between 2000–2007, compared survival outcomes between 8,289 DBD kidneys and 845 DCD kidneys (25). Despite increased rates of delayed graft function (DGF) after DCD kidney transplantation, first-time recipients of DCD kidneys (*n* = 739) or DBD kidneys (*n* = 6,759) showed no difference in 5-year graft survival (HR 1.01, 95% CI 0.83–1.19, *p* = 0.97). Increasing donor or recipient age, repeat transplantation, and CIT >12 h were associated with worse graft survival for recipients of DCD kidneys. Subsequent analyzes demonstrate equivalent 5-year patient survival or 10-year death-censored graft survival comparing DCD versus DBD kidneys (26). Prolonged CIT (>24 h versus <12 h) was associated with poorer graft survival for DCD versus DBD kidneys in cohorts from the United Kingdom and United States (27). The rate of primary nonfunction for both DCD and DBD kidneys was low (3.1 and 2.5% respectively) and not significantly different (risk-adjusted OR 1.18, 95% CI 0.9–1.5, *p* = 0.21) (28). These reassuring long-term outcomes suggest DCD kidneys of any age should be actively considered for all kidney transplant candidates, if logistics and resources can facilitate timely surgery to avoid prolonged CIT.

Studies report heterogeneous outcomes for ECD-DCD kidneys. Locke et al., exploring data from the United States between 1993–2005, observed donor age was associated with increased graft failure risk, although graft survival was similar between ECD-DBD and >50-year old DCD kidneys (29). Singh et al., analyzing data from the United States including 562 ECD-DCD kidneys, showed slightly increased risk for graft loss in recipients receiving DCD versus non-DCD kidneys, which was not significantly modified by ECD status (30). Across a number of studies, ECD-DCD kidneys report acceptable 3-year death-censored graft survival rates between 70%–90%, which are inferior to SCD-DCD kidneys but not ECD-DBD kidneys (26-32). However, Montero et al. demonstrate how important selecting the most appropriate donor-recipient combination is in a recently published risk modelling study (33). In their multi-center cohort study, mortality risk for the highest risk-stratification group receiving ECD-DCD kidneys was significant. Although survival was better post-transplantation compared to remaining waitlisted, it raises a level of caution in decision making when dealing with donor-recipient extremes. Therefore, use of ECD-DCD kidneys is acceptable for select waitlisted kidney transplant candidates when carefully balanced against their mortality risk without transplantation and quality of life considerations.

### Kidney Donor Risk Index

The KDRI is a risk quantification score developed in 2009 by Rao et al. using data from the United States between 1995–2005 containing 14 donor- or transplant-specific variables (34). A recent re-evaluation using more contemporary United States data reported the original KDRI remains robust for discrimination and predictive accuracy for graft failure (35). KDRI has been implemented into allocation policy within the United States, with low KDRI (i.e., better quality) kidneys preferentially allocated to kidney transplant candidates with the greatest expected longevity (36).

A pan European study including 24,177 adult kidney transplant recipients demonstrated an increase in KDRI by 1.3% annually, from 1.31 (IQR 1.08–1.63) in 2005 to 1.47 (IQR 1.16–1.90) in 2015, driven by increased donor age, hypertension, and use of DCD kidneys (16). No difference was observed in 5-year patient or allograft survival outcomes, with survival probabilities improving over time for the highest KDRI kidneys. Within any given KDRI interval, although ECD kidneys have higher rates of discard and graft failure risk, the ECD categorization does not confer additional risk of discard or graft failure compared with SCD kidneys within the same KDRI interval (37).

However, caution should be exercised with the KDRI. It contains components which can increase the risk quantification score but now demonstrate comparable outcomes (e.g., DCD). Translatability of the KDRI to population cohorts outside the United States may not be valid (38,39). Due to disparate survival outcomes observed for kidney failure patients treated with dialysis (40,41) versus kidney transplantation (42) in the United States versus elsewhere, and different utilization of deceased donors (e.g., greater use of older and DCD kidneys in the United Kingdom versus the United States for example) (43), generalizability may be invalid.

### Donor Age

Donor age has the strongest independent association with long-term kidney transplant outcomes (44). These accepted deleterious effects justify donor age being a component of the KDRI risk score but also the fundamental stratification for ECD classification. Donor age is one of the most frequent explanations for organ discard (8), despite an increasing proportion of deceased organs over time being procured from older donors (16). While many studies dichotomize at an arbitrary cut-off donor age of 60 years, deleterious effects for kidney transplant recipients may start earlier. Keith et al. analyzed data from the United States between 1990 and 1997 and observed adjusted 10-year patient survival starts to drop with deceased donor ages 36–40 years (45). There is a strong interaction between donor and recipient age, with additive detrimental effect on allograft survival with a combination of older kidneys into older recipients (46), although many allocation systems prioritize on this like-for-like basis.

Some centers consider dual versus single kidney transplants using older kidneys. However, when using donors aged ≥60 years, no graft survival advantage at 5-year was observed comparing dual versus single kidney transplantation in an analysis from the United Kingdom between 2005–2017 (adjusted HR 0.81, 95% CI 0.59–1.12). However, dual kidney transplantation did result in slightly higher 1-year estimated glomerular filtration rate [eGFR] (40 versus 36 ml/min/1.73 m^2^ respectively, *p* < 0.001) (47).

### Donor Ethnicity

Non-white ethnicity demonstrates conflicting associations with kidney transplantation outcomes. Pisavadia et al., exploring data from the United Kingdom between 2003–2015, observed higher risk for graft loss with south Asian (HR 1.38, 95% CI 1.12–1.70, *p* = 0.003) and Black (HR 1.66, 95% CI 1.30–2.11, *p* < 0.001) donated kidneys independent of recipient ethnicity, with no survival advantage from donor-recipient ethnicity matching (48). Locke et al., exploring data from the United States between 1993–2006, suggested DCD kidneys from Black donors, but not DBD kidneys, were associated with better patient and graft survival for Black recipients (49). This contrasts with evidence from registry data that kidneys donated by ethnic minorities (especially Black individuals) are associated with poorer graft survival for any kidney transplant recipient (50,51).

However, using ethnicity for risk stratification of deceased donors is questionable. Ethnicity is not a reliable proxy for genetic difference between individuals (52). While incorporating ethnicity into clinical decision-making can be considered a form of personalized medicine, it may not add additional value. For example, Chong et al., in an analysis of data from the United States between 2000–2017, demonstrated removal of donor ethnicity from KDRI calculations makes negligible difference to patient and kidney allograft survival, strongly advocating for removal of donor ethnicity as a risk factor (53).

### Donor Body Mass Index

In a population cohort study from the United Kingdom, Arshad et al. observed an independent association between donor BMI and delayed graft function (54), with risk increased in recipients of kidneys from overweight (Odds Ratio [OR] 1.12, 95% CI 1.00–1.23, *p* = 0.022), obese (OR 1.23, 95% CI 1.08–1.39, *p* < 0.001), and morbidly obese (OR 1.38, 95% CI 1.16–1.63, *p* < 0.001) donors when compared to normal donor BMI group. However, donor BMI did not influence long-term patient or graft survival. This is corroborated with data from the United States. In a study of 6,932 recipients of DCD kidneys in the United States, Ortiz et al. reported donors with a BMI between 30.0–34.9 kg/m^2^ incurred 1.77-fold increased odds of developing DGF, with similar odds for DGF in donors with a BMI between 35.0–39.9 kg/m^2^ (OR 1.78, *p* < 0.001) (55). However, only DCD kidneys from donors with a BMI >45.0 kg/m^2^ were associated with an increased risk of death-censored graft failure (adjusted HR 1.84, 95% CI 1.23–2.74, *p* < 0.001) relative to a normal donor BMI category.

### Donor Size

The influence of donor-to-recipient size matching has shown conflicting results. Arshad et al., exploring data from the United Kingdom between 2003–2015, showed no association between donor-to-recipient size match difference and risk for DGF or death-censored graft survival (56). Donor-to-recipient difference in body weight was associated with higher 12-month creatinine in large recipients receiving small donor kidneys. Increased mortality was observed in recipients receiving larger kidneys (HR 1·21, 95% CI 1.05–1.40 *p* = 0.009), which conflicts with other population-cohort studies that show inferior long-term patient and graft survival associated with larger recipients receiving smaller donor kidneys (57-59). Some show negative effects of size mismatch (large kidney into small recipient) only in the context of ECD kidneys (58) or male recipients of female kidneys (59).

### Donor Acute Kidney Injury

The relationship between donor AKI and kidney transplant outcomes has been reviewed by Koyawala and Parikh (60). In total, 37 studies were identified comparing transplant outcomes between kidneys with versus without donor AKI. Donor AKI was associated with DGF, with prolonged nights in hospitals and additional attributed costs. In a separate meta-analysis of 14 cohort studies exploring 15,345 donors, Zheng et al. estimate the relative risk of DGF to be 1.76 (95% CI 1.52–2.04) for recipients of kidneys with versus without donor AKI (61).

No association is seen between donor AKI and risk for rejection after 6 months or 1 year, either in a review of published studies (60) or meta-analysis of empirical data (RR 0.87, 95% CI 0.66–1.15) (61). No association was seen between donor AKI and graft function (60).

From a graft survival perspective, donor AKI was not associated with graft failure in 25/29 studies (60). However, some studies provide more granular insight. Botha et al. analyzed 11,219 transplanted kidneys in the United Kingdom, comprising 1,869 (17%) with AKI (62). While 1-year graft survival difference was statistically significant comparing AKI versus non-AKI donor kidneys, the numerical difference was clinically insignificant (89% versus 91% respectively, *p* = 0.02). DGF rates increased with severity of AKI (no AKI = 28%, AKI stage 1 = 35%, AKI stage 2 = 43%, AKI stage 3 = 55%, *p* < 0.005). Primary nonfunction rates were higher with donor AKI stage 3 versus no AKI kidneys (9% versus 4%, *p* = 0.04) and graft function was lower among donor AKI kidneys (OR 1.25, 95% CI 1.08–1.31, *p* < 0.005). This study differed from other cohorts due to its higher sample size, with a larger proportion of donor kidneys with severe AKI and donation after circulatory death, meaning this study may be better powered to observe differences in outcomes among donor kidneys with higher levels of injury. Other studies observed higher rates of graft failure only among a sub-select of studies using ECD donor kidneys with AKI (63,64).

Donor AKI is more acceptable with high versus low quality kidneys. Single center outcomes using donors with both AKI (comparing advanced stages 2–3 versus 0–1) and high KDPI (≥85%) demonstrated more DGF (71% versus 37% respectively, *p* < 0.001), more primary nonfunction (5.3% versus 0.6% respectively, *p* = 0.02), no difference in eGFR in ml/min/1.73^2^ (44 versus 46 respectively, *p* = 0.42) and lower 1-year death-censored graft failure 14.5% versus 3.5% for AKI 2-3 versus AKI 0-1 high KDPI kidneys respectively (HR 2.40, 95% CI 1.24–4.63, *p* = 0.01) (65).

### Donor Diabetes

Cohen et al. studied survival outcomes for kidney transplant patients receiving diabetic versus non-diabetic kidney allografts in the United States between 1994–2013 (66). Recipients of diabetic donor kidneys had higher rates of all-cause allograft failure (HR 1.21, 95% CI 1.16–1.26) and death (HR 1.19, 95% CI 1.13–1.24) compared to receiving non-diabetic donor kidneys. Allograft survival was worse for younger (≤45 years of age) versus older recipients of diabetic donor kidneys, but no difference was observed in patient survival. Due to a significant interaction between donor and recipient diabetes status (with diabetic recipients receiving diabetic donor kidneys having the worst patient and allograft survival), paired analyzes of mate-kidneys from the same donor were undertaken where one recipient was diabetic and the other non-diabetic. In this analysis, diabetic recipients had significantly higher risk of allograft failure (HR 1.27, 95% CI 1.05–1.53) and death (HR 1.53, 95% CI 1.22–1.93) compared to non-diabetic recipients. Diabetic recipients of non-diabetic donor kidneys and non-diabetic recipients of diabetic donor kidneys had similar rates of all-cause allograft survival.

The critical question is whether waitlisted patients should accept diabetic donor kidneys versus waiting for better kidneys. Cohen et al. compared survival benefits of kidney transplantation using diabetic donor kidneys versus remaining on the waiting-list in the United States between 1994–2015 (67). They observed recipients of diabetic donor kidneys had lower mortality compared with remaining on the waiting-list and/or transplantation later with a non-diabetic donor kidney (adjusted HR 0.91, 95% CI 0.84–0.98). Although recipients of non-diabetic donor kidneys with high KDPI scores had lower mortality risk (adjusted HR 0.86, 95% CI 0.81–0.91), recipients of diabetic donor kidneys with similar high KDPI scores showed no survival difference (adjusted HR 1.09, 95% CI 0.97–1.22). Younger waitlisted patients (aged <40 years) had no survival benefit from transplantation with diabetic donor kidneys, while diabetic patients with longer waiting-list times attained the greatest survival benefit.

### Donor Hypertension

Donor hypertension is increasing in prevalence and observed in nearly a third of deceased donors (16). Altheaby et al., in a systematic review and meta-analysis, identified 15 studies published between 1963–2014 exploring the association between donor hypertension and kidney transplant outcomes (68). Pooled risk ratios (RR) demonstrate donor hypertension is associated with kidney allograft failure (RR 1.31, 95% CI 1.06–1.63, *p* = 0.014) but not mortality (RR 0.996, 95% CI 0.652–1.519, *p* = 0.984).

### Donor Smoking

Donor smoking and kidney transplant outcome associations are unclear. Lin et al. explored data from the United States between 1994–1999 and observed smoking history among deceased kidney donors was associated with increased transplant recipient risk for death and graft loss (69). However, Gillott et al. explored data from the United Kingdom between 2001–2013 and observed no association between donor smoking and allograft survival for kidney transplant recipients, although an association with mortality was observed (adjusted HR 1.16, 95% CI 1.03–1.29, *p* = 0.011) (70).

### Donor Cause of Death

Death by cerebrovascular accident remains the commonest cause of death, varying little across Europe between 2005–2015 (16), and contributes to ECD classification for donors aged ≥50 years. Few studies have explored the impact of cause of donor death and recipient outcomes, although cause of death that can result in disease transmission has been of greater concern.

### Donor-Derived Disease Transmission

Risk for donor-derived disease transmission (defined as either infection or malignancy) leading to morbidity or mortality occurred in only 0.96% of all solid organ transplantation in the United States (71). Increased risk for disease transmission (IRD) kidneys tend to be better quality (defined as lower KDPI scores) and associated with survival benefits. For example, Bowring et al. analyzed data in the United States between 2010–2014 and demonstrated: 1) recipients who declined IRD kidneys and subsequently received non-IRD kidneys accepted a higher median KDPI (21 versus 52 respectively); and 2) after a short risk period in the first 30 days following IRD acceptance (adjusted HR 2.06, 95% CI 1.22–3.49, *p* = 0.008) (absolute mortality 0.8% versus 0.4%), those who accepted IRDs had lower risk of death 1–6 months (adjusted HR 0.67, 95% CI 0.50–0.90, *p* = 0.006) and beyond 6 months (adjusted HR 0.52, 95% CI 0.46–0.58, *p* < 0.001) (72).

However, most cases of donor-disease transmission will occur in clinically covert donors. For example, in a systematic review of published literature, donors with a history of cancer or an ongoing malignancy contributed to disease transmission in only 17.1% (*n* = 32) of cases (73). Using data from the United Kingdom, it is estimated the risk of transmitting cancer from a donor not known to have a malignancy is very low at 1 in 2,000 (0.05%) (74).

### Donor Increased Risk Behavior

Increased risk behavior (IRB) among deceased donors can be classed as intravenous drug use (IVDU), imprisonment, or high-risk sexual behavior. Trotter et al, analyzing data from the United Kingdom between 2003–2015, studied the outcomes associated with use of IRB deceased donor kidneys (75). Donors with IRB provided 1,091 organs for transplantation (including 624 kidneys) and transplant outcomes were similar in recipients of organs from donors with versus without IRB. Only three cases of unexpected hepatitis C virus transmission were identified, all from an active IVDU donor who was hepatitis C virus seronegative at time of donation but was found to be viremic on retrospective testing. National registry data and single center studies from the United States have shown excellent outcomes and minimal risk associated with using deceased donor kidneys from IVDU individuals (76-79). High decline rates observed with IRB kidneys (75,76) suggest a valuable but underutilized resource due to a subjective perception of heightened risk for kidney transplant recipients not supported by objective evidence.

## Donor Histopathology

The benefit of obtaining donor histopathology to guide kidney utilization is unclear. In a systematic review of published evidence, Wang et al. combined empirical evidence from 47 studies (80). In these retrospective studies exploring heterogenous histopathological criteria, no semi-quantitative scoring system was conclusively associated with post-transplant outcomes including DGF, grant function, and/or graft failure. This may relate to weak inter-observer correlation and variability between pathologists, which could be improved using a dedicated pool of specialist pathologists (81). Preimplantation biopsy analysis may be useful in a subset of deceased donor kidneys where chronic injury is prevalent like ECD kidneys. Based upon this rationale, the PreImplantation Trial of Histopathology In renal Allografts (PITHIA) study is an open, multicenter, stepped-wedge cluster randomized study, that involved all UK adult kidney transplant centres (82). Using a pool of dedicated pathologists, it will explore whether a national, 24-h, digital histopathology service improves organ utilization from deceased donors aged 60 years and over. The results from this national study are awaited but should provide clarity regarding the value of pre-implantation donor histopathology.

## Decision Challenges

**FIGURE 3:**
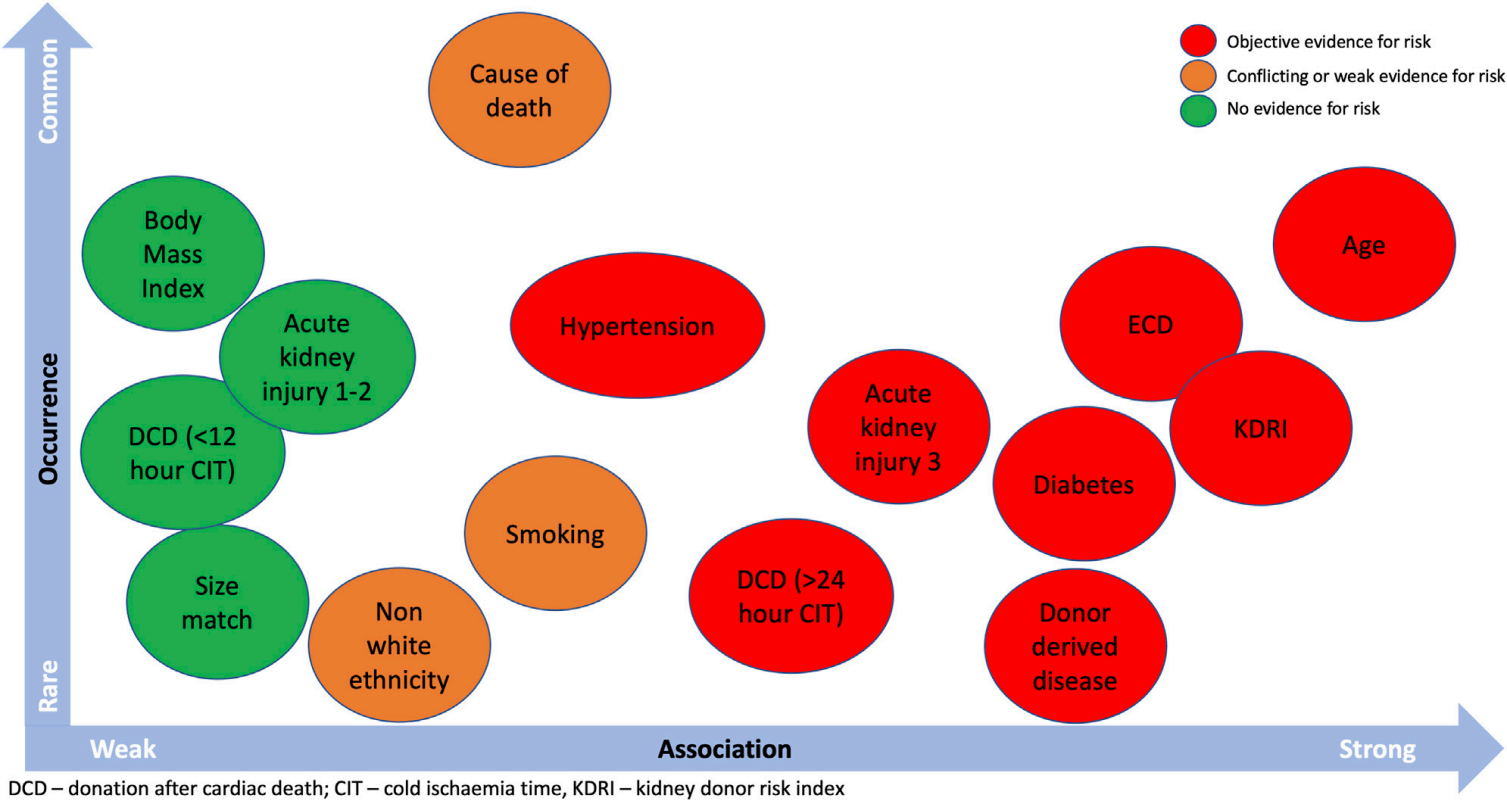
Donor characteristics that can influence kidney allograft outcomes and the probability of occurrence.

Translating this evidence to nuanced decision-making is the big challenge. No guidelines or recommendations exist to support this process, which is difficult considering the nature of available data. For example, most deceased donors will have a combination rather than individual clinical factors (see Figure 3). This requires individualized considerations of population-level data which are not amenable to simple flowcharts. Organ utilization has behavioral components, from both patients and professionals, that will influence decision-making, and it is important every kidney transplant candidate receives the same opportunities (83). Therefore, consensus recommendations to support decision-making may be welcomed by the transplant community.

However, this is a challenge due to the multi-factorial factors that influence post-transplant outcomes. Kerr et al., exploring data from the United States, quantified the magnitude of paired deceased donor effects when transplanted into different recipients (84). In analyses adjusted for KDPI, Kerr et al. demonstrated moderate donor effects for DGF and minimal donor effects for 1- and 3-year graft failure, with 4%–8% excess absolute risk over baseline for a graft if the mate kidney failed. Therefore, it is important to appreciate that post kidney transplant outcomes are influenced by a complex interplay of factors that include, but are not exclusive to, donor characteristics.

Developing and validating novel strategies and/or techniques to improve the process is therefore necessary. Various tools to aid decision-making are currently available or under investigation. These include donor risk scores in the setting of DCD kidneys (85), donor-recipient characteristics (86), donor-specific features (87), monitoring of perfusion parameters and assessment of tissue viability function *ex situ* (88), molecular diagnostics (89), and machine learning and artificial intelligence (AI) algorithms (90-92). The latter remains in its infancy, with tremendous potential to augment the decision-making regarding transplantation (93), but requires more granular data, generalizability, and validation across different population cohorts to enter mainstream use. Such AI tools must provide survival probabilities for kidney transplant candidates to proceed with an individual organ offer versus remaining on the waiting-list to allow a meaningful decision to be made about transplantation. While some risk communication tools are available (http://www.transplantmodels.com or https://www.odt.nhs.uk/transplantation/tools-policies-and-guidance/risk-communication-tools/for the US and UK respectively), they lack the machine learning capability or enhanced AI to provide more personalized risk probabilities.

## Conclusion

Complex deceased donor kidney offers, with time-pressured decision-making, can lead to unnecessary decline and/or discard of acceptable kidneys. By outlining donor clinical factors associated with post kidney transplant outcomes, the aim of this review is to support clinical decision-making. However, donor characteristics are only one component of a complex interplay that influence post-transplant outcomes. While any kidney allograft may not be better than no kidney allograft in every clinical scenario, the objective evidence would argue most kidney allografts are better than being denied the opportunity of kidney transplantation if deemed suitable for waitlisting.
